# Development of a *Providencia stuartii* multilocus sequence typing scheme

**DOI:** 10.3389/fmicb.2024.1493621

**Published:** 2024-10-31

**Authors:** Gabriele Arcari, Alice De Francesco, Riccardo Polani, Alessandra Carattoli, Valerio Capitani

**Affiliations:** ^1^Department of Medicine and Technological Innovation, University of Insubria, Varese, Italy; ^2^Ospedale di Circolo e Fondazione Macchi, Laboratory of Medical Microbiology and Virology, Varese, Italy; ^3^Department of Molecular Medicine, Sapienza University of Rome, Rome, Italy; ^4^Department of Public Health and Infectious Diseases, Sapienza University of Rome, Rome, Italy

**Keywords:** *Providencia stuartii*, MLST, phylogenomics, gene, allele

## Abstract

**Introduction:**

The *Providencia* genus is assuming greater clinical relevance among infections caused by *Enterobacterales* also because of its intrinsic and acquired resistance to last-resort antibiotics. However, despite having been known and studied for over 50 years, genomics and taxonomy of the *Providencia* genus are currently undergoing a deep rearrangement. In this study we aim to outline and characterized the *P. stuartii* species.

**Methods:**

We retrieved from the GenBank database all genomes labelled as *Providencia* and performed a comprehensive genome-based species definition founded on average nucleotide identity (ANI) and on alignment-free approaches.

**Results:**

After defining the genomes assuredly identifiable as *P. stuartii*, we devised a MultiLocus Sequence Typing (MLST) and a core-genome MLST (cgMLST) schemes, based on 7 and 2,296 loci respectively.

**Discussion:**

This work hence provides a framework for understanding the role of *P. stuartii* and of other members of this genus, which should be considered as emerging multidrug-resistant pathogens.

## Introduction

The genus *Providencia* has traditionally been classified as part of the *Proteeae* tribe (Ewing, 1962). Nonetheless, based on a recent proposal for the classification of genera previously assigned to the *Enterobacteriaceae* family, this genus is now recognized as a member of the *Morganellaceae* family within the *Enterobacterales* order (Adeolu et al., 2016).

Strains belonging to the family *Morganellaceae* have a significant impact not only as environmental bacteria (Ferheen et al., 2023) or opportunistic pathogens (Liu et al., 2016) but also as infectious agents (Salas et al., 2023) in both human (Minnullina et al., 2019) and animal settings (Palmieri et al., 2020). In fact, members of this family are characterized by virulence traits, such as biofilm formation (Hola et al., 2012), motility, and cell invasion (Kurmasheva et al., 2018).

These virulence traits are invariably coupled with intrinsic resistance to several last-resort antibiotics such as colistin [due to the carriage of the *arnBCADTEF* and *eptB* genes (Gogry et al., 2021)], tigecycline (Yaghoubi et al., 2022), and imipenem (Clinical and Laboratory Standards Institute, 2024) and, occasionally, with acquired resistance genes [mostly *bla*_NDM_ and *bla*_VIM_ (Mondol et al., 2024; Abdallah and Balshi, 2018)]. Hence, the use of these antimicrobial agents to treat multidrug-resistant (Magiorakos et al., 2012) strains is significantly promoting the spread of members of the family *Morganellaceae* (Hayakawa et al., 2012) through bystander selection (Tedijanto et al., 2018).

As of June 2023, the genus *Providencia* accounted for 11 different recognized species such as *Providencia alcalifaciens, Providencia burhodogranariea, Providencia heimbachae, Providencia manganoxydans, Providencia rettgeri, Providencia rustigianii, Providencia sneebia, Providencia stuartii, Providencia thailandensis, Providencia vermicola,* and *Providencia huaxiensis,* and a twelfth species, the candidatus *Providencia siddallii*, was under evaluation. However, the taxonomy of *Providencia* is an ever-changing field, and, as of June 2024, the *P. thailandensis* species has been redefined as *P. stuartii* (Dong et al., 2024) and two novel species, namely, *Providencia hangzhouensis* (Dong et al., 2023) and *Providencia zhijiangensis* (Dong et al., 2024), were enlisted among the members of this genus.^1^ Of these species, *P. alcalifaciens*, *P. rettgeri,* and *P. stuartii* are the most notorious since they are more frequently ascribed to clinically relevant events (Mnif et al., 2013; Shah et al., 2019; Liu et al., 2023). However, owing to the contemporary genomic-based revision of the genus *Providencia,* its taxonomy is undergoing several extensive refinements, which highlighted the presence of species yet to be clearly defined (Wu et al., 2023; Yuan et al., 2020).

In this context, a major change is the above-mentioned proposal to consider “*Providencia thailandensis*” as a heterotypic synonym of “*Providencia stuartii*.” Indeed, the two type strains of these species display a low average nucleotide identity (ANI) difference (Dong et al., 2024). Under an epidemiology point of view, this change increased the relevance of *P. stuartii* and emphasized the necessity of a typing scheme for this species.

In this manuscript, we started by outlining the genomes that should be attributed to *P. stuartii* (i.e., displaying an ANI > 95% with the reference genome) and developed a core-genome multilocus sequence typing and a 7-gene multilocus sequence typing for this species.

## Materials and methods

### Genome retrieval and quality evaluation

As of 1 June 2022, the NCBI genome downloading script (*ncbi-genome-download bacteria --genera Providencia --assembly-levels all --formats fasta*)^2^ was used to download all (386) genomes from isolates identified as *Providencia* species. To these, three genomes sequenced in a previous study were added (Capitani et al., 2023) for a total of 389. The only genome assemblies of *Providencia manganoxydans* and of the candidatus *Providencia siddallii* were discarded, being the first contaminated and the latter partial.

The quality of the remaining 387 genomes was assessed by the BUSCO tool version 5.3.2 (Simão et al., 2015), using the Enterobacterales_odb10 set created on 23 February 2021. A total of 7 genomes displaying a BUSCO score lower than 95% were excluded from further analyses, resulting in a 380 genome dataset.

### Evaluation of the taxogenomic parameters

Genome relatedness between the *P. stuartii* type strain genome (NCBI RefSeq assembly no. GCF_029075745.1, isolate ATCC 29914) and the *Providencia* genome was assessed by ANI using the CompareM tool v0.0.23.^3^

### Phylogeny and population structure analyses

The genome labeled as *P. stuartii* according to ANI was annotated using Prokka (Seemann, 2014), and the resulting. gff files were used as input for Roary (Page et al., 2015). A core-genome alignment was generated using standard Roary parameters (95% identity for BLASTp and presence in 99% of isolates to be defined as “core”) and used to infer a phylogenetic tree using the IQ-TREE (Minh et al., 2020) [GTR + F + R4 model (Kalyaanamoorthy et al., 2017)] and 10,000 ultrafast bootstraps (Hoang et al., 2018).

This procedure was repeated twice with the same parameters using the two *P. stuartii* datasets (1 June 2022 and 23 June 2023).

The POPulation Partitioning Using Nucleotide Kmers (PopPUNK) tool version 2.5.0 (Lees et al., 2019) was used to analyze the relative pairwise genetic distances of the core (*π*) and accessory (a) genomes and infer the population structure of *Providencia*. The PopPUNK quality control, using a π distance of 0.17, a distance of 0.9, and a length differing of 3 or more standard deviations from the mean as thresholds, discarded 43 isolates. A refined dbscan fit model was deployed and visualized using Cytoscape (version 3.10.2).

The final versions of the PopPUNK dbscan graph and of the phylogenetic trees were adjusted using the open-source InkScape software (version 1.3.2).

### Construction of the cgMLST and MLST schemes

A core-genome multilocus sequence typing (cgMLST) and MLST schemes were devised for the *P. stuartii* species complex. Specifically, the cgMLST scheme was developed using the chewBBACA tool (Silva et al., 2018) on a subset of 51 *P. stuartii* genomes [48 submitted to the GenBank database as of 1 June 2022, and 3 genomes sequenced in a previous study (Capitani et al., 2023)].

Single-nucleotide polymorphisms (SNPs, determined using the SNP distance tool matrix)^4^ on the core genes among genomes of the subset were used to define the different sequence types (STs).

We checked the selected seven MLST loci for homoplasy with SplitsTree (Huson and Bryant, 2006), assessed the presence of potential selection pressure with the FUBAR, aBSREL, SLAC, and BUSTED tools from the HyPhy suite (Kosakovsky Pond et al., 2020), calculated Tajima’s D and dN/dS ratio with MEGA (Oxford academic, 2021) and evaluated several additional genomic metrics (i.e., haplotype diversity, nucleotide diversity, and segregating sites) with DnaSP6 (Rozas et al., 2017). Finally, single-linkage hierarchical clustering of the cgMLST alleles was performed across various thresholds (5, 10, 50, 100, 250, 500, 750, and 1,000) using Reportree (Mixão et al., 2023), and all were compared with the seven-loci MLST scheme using the Comparing Partitions online tool (Carriço et al., 2006) to define Simpson’s diversity index and estimating a 95% confidence interval using jackknife (Severiano et al., 2011).

### *In silico* validation of *Providencia stuartii* MLST loci and scheme

As of 23 June 2023, the NCBI genome downloading script was used again with the same parameters as of 1 June 2022 (*ncbi-genome-download bacteria --genera Providencia --assembly-levels all --formats fasta*), and 202 more genomes were downloaded. The quality of these remaining 202 genomes was assessed by the BUSCO tool version 5.3.2 (Simão et al., 2015), and no genome was excluded from further analyses according to its BUSCO score.

Out of the 202 novel *Providencia* genomes submitted in GenBank between 1 June 2022 and 23 June 2023, we identified 26 genomes displaying an ANI > 95% with the *P. stuartii* reference genome and hence attributable to this species. These genomes were used to assess *in silico* the newly devised MLST scheme.

### Experimental validation of the *Providencia stuartii* MLST scheme

We also developed a PCR-based MLST scheme based on seven housekeeping genes to be applied when genomic facilities are not available. Amplification reactions contained 3 U Taq DNA polymerase (Meridian Bioscience, Cincinnati, OH, United States of America), 10 μL 5× PCR buffer, 2 μL amplification primers (10 μM each, Supplementary Table S1), 34 μL deionized H_2_O, and 1.5 μL bacterial template. We obtained bacterial template by the boiled DNA from a fully sequenced *P. stuartii* isolate [isolate 41, genome acc. No. CP142095 (Capitani et al., 2023)]. Amplification was performed starting with 2 min at 95°C to activate the DNA polymerase, followed by 29 cycles consisting of denaturation (94°C, 25 s), annealing (55°C, 25 s), and extension (72°C, 1 min and 15 s, Supplementary Table S1).

Amplicons were analyzed using Sanger sequencing. While most genes were sequenced using amplification primers, the *znuA* amplicon (owing to its size and to the distance of allele-defining SNPs from the primers) needed two additional primers (znuA_seq_1 and znuA_seq_2) specifically for sequencing the variable region (Supplementary Table S1). The resulting sequences were then aligned to the corresponding *P. stuartii* allele using BLASTn.

## Results and discussion

### Cluster distribution of the genus *Providencia* genomes

Recent genomic analyses offered a detailed classification of species and lineages belonging to the genus *Providencia*, highlighting a wide species diversity (Dong et al., 2024; Wang et al., 2023).

**Figure 1:**
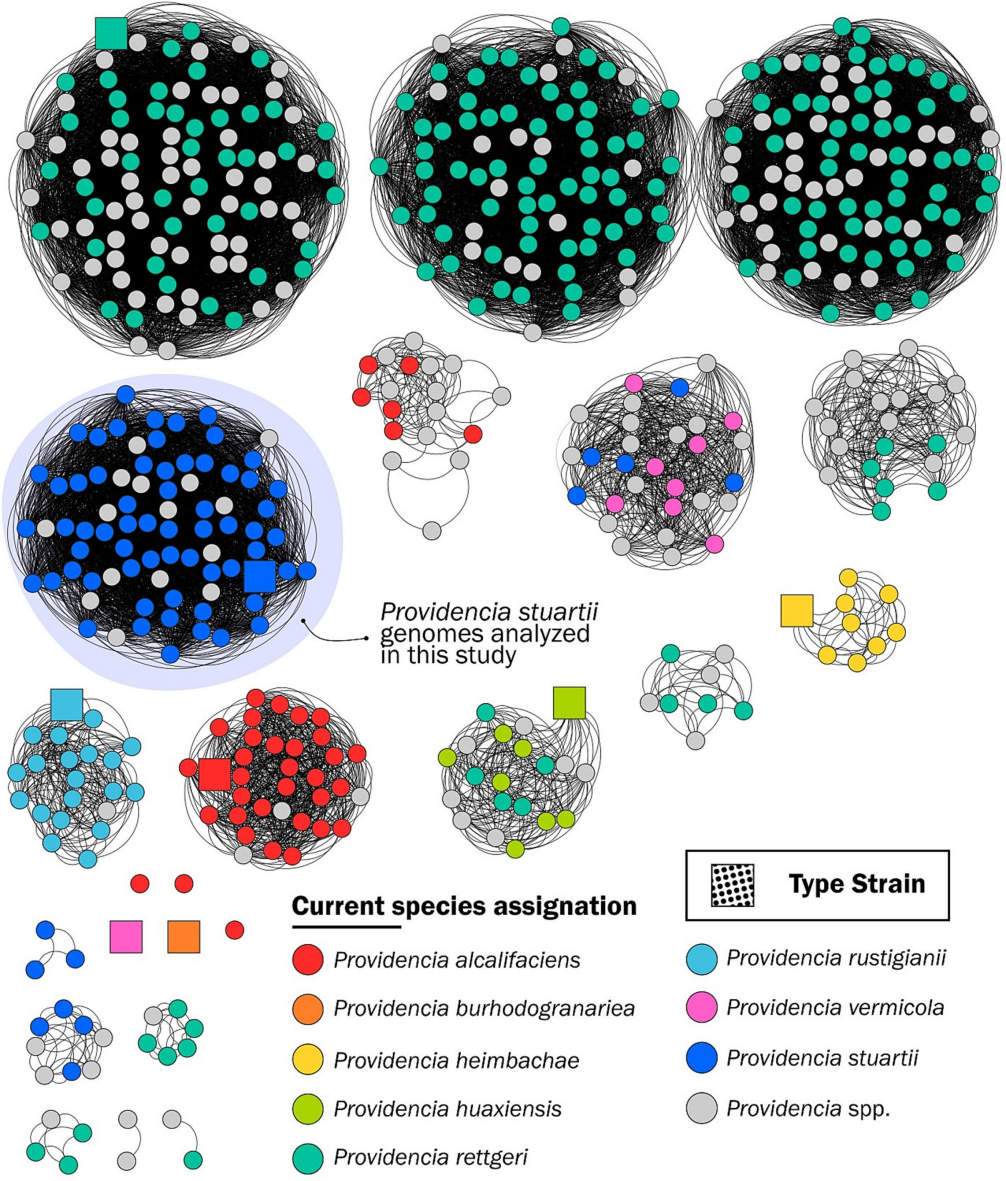
Grouping of 581 *Providencia* spp. genomes downloaded from the GenBank database as of 23 June 2023, generated by PopPUNK (Lees et al., 2019). The nodes are color-coded according to the legend and indicate the current NCBI assignation of the genome; square nodes represent the reference strain genomes.

A total of 335 *Providencia* genomes (386 from the GenBank database as of 23 June 2023 and 3 from our collection, deprived of 7 genomes displaying a low BUSCO score, the *P. manganoxydans* and the *P. siddallii* reference genomes, and 43 genomes that did not pass the PopPUNK quality module) were included in the PopPUNK population analysis. These genomes defined 23 clusters (5 of which constituted only by one singleton) (Lees et al., 2019) (Figure 1). Two of the singletons were the *P. burhodogranariea* and *P. vermicola* reference strain genomes. Of note, all other eight genomes submitted in GenBank as *P. vermicola* clustered with five genomes labeled as *P. stuartii* and with genomes of unclassified *Providencia* spp.

The *P. heimbachae* and *P. rustigianii* clusters contain genomes that are coherently assigned to the respective *Providencia* species. Genomes assigned to *P. rettgeri* exhibit the highest rate of potential misclassification, with a dispersed distribution in nine different clusters, where only one comprising the *P. rettgeri* reference genome strain.

Since the *P. zhijiangensis* and the *P. hangzhouensis* genomes were released in November and September 2023, respectively, they were not included in this analysis.

### Definition of a cgMLST scheme and selection of MLST loci

We devised a specific cgMLST for the *P. stuartii* species complex. This was based on 51 genomes downloaded from the GenBank database as of 1 June 2022, displaying an ANI > 95% with the *P. stuartii* genome of the reference strain (NCBI RefSeq assembly no. GCF_029075745.1, isolate ATCC 29914). Out of these 51 genomes, 3 were from a previous study (Capitani et al., 2023) and 48 were retrieved from the NCBI RefSeq database, not taking into consideration if they were submitted in GenBank as *P. stuartii* or *Providencia* spp.

The chewBBACA tool version 3.2.0 (Silva et al., 2018) was deployed, using the NCBI RefSeq assembly no. GCF_029075745.1 (isolate ATCC 29914) as a training file. After the removal of 1,594 loci (32 containing ambiguous or invalid characters and 1,562 shorter than 201 bp), a total of 7,561 loci were identified, showing a total of 53,725 alleles. Of these loci, 2,296 corresponded to the genes of the cgMLST (identified as present in every isolate).

The SNP distance matrix generated starting from the Roary core-genome alignment displayed either <1,100 SNPs or > 7,500 SNPs; hence, we defined each *P. stuartii* ST as a group of genomes showing less than 1,100 SNPs in the apart one from the other (Supplementary Dataset 1).

The 7-gene MLST was determined starting from the cgMLST alleles as determined by chewBBACA (Silva et al., 2018). The *P. stuartii* SNP-based phylogenetic tree on the core genome from the 51 genomes displayed a comb-like disposition with 22 branches (Supplementary Figure S1). Each branch corresponded to one of the 22 STs above defined.

After removal of the genes whose alleles did not fit with the 22 assigned ST, 471 loci remained. To ensure the stability and the discriminatory power of the MLST scheme, only 184 loci displaying more than 4 but less than 10 different alleles were considered. Of these, a representative of each protein was annotated using the UniprotFinder module of chewBBACA (Silva et al., 2018). After removal of genes coding for hypothetical or not fully characterizable proteins, non-structural proteins (e.g., fimbriae), and subjectable to selective pressure (e.g., porins or antimicrobial efflux systems), 15 suitable candidate genes were selected.

To assess species specificity, we performed a BLASTn screening of the 15 candidate genes against the *Providencia* genus collection. These genes showed a nucleotide identity >99.9% only with genomes displaying an ANI > 95% with the *P. stuartii* reference genome, whereas their identity dropped to <90% with other *Providencia* species.

Finally, using Bandage 0.8.1 (Wick et al., 2015), we mapped these genes on the circular ST3 *P. stuartii* genome 41 (genome acc. No. CP142095) to assess their distribution alongside the genome (Supplementary Figure S2). Using the gene position criterion to minimize the risk that a single recombination event would affect multiple genes, we limited the number of genes to seven.

The proteins encoded by the genes chosen as representatives of the MLST scheme are as follows:

- GreA, encoding a transcription elongation factor that is highly conserved among prokaryotes and that regulates gene expression (Yuzenkova et al., 2014; Feng et al., 2020).
- FtsH, a protease involved in the cell division process (Ito and Akiyama, 2005).
- TolR, the stator protein of the Tol-Pal system, is involved in the outer membrane invagination during cell division (Wojdyla et al., 2015).
- ArnE, part of the ArnE/ArnF heterodimer, by transporting the 4-amino-4-deoxy-L-arabinose (L-Ara4N) is involved in lipid A modification (Yan et al., 2007).
- ZnuA, responsible (together with ZnuB and ZnuC) for the intracellular Zn^++^ homeostasis (Ammendola et al., 2007).
- YciA, a hexameric broad specificity acyl-CoA thioesterase plausibly involved in membrane biogenesis (Willis et al., 2008).
- RseA, an anti-sigma-E factor protein that averts the transcription of the sigma-E factor induced by heat-shock promoters (de Las Peñas et al., 1997).

We performed multiple tests to assess the quality of the chosen loci. Positive selection examinations did not report any significant positive selection on all branches, and no loci displayed a dN/dS ratio greater than 1 (Supplementary Dataset 1). Bayesian analyses confirmed that most codon positions in all seven genes are neither positively nor negatively selected, with six of the seven MLST genes displaying a variable number of codons conserved under purifying selection with low Bayes factor values (ranging from 0.008 to 0.093), except for codon 16 in the *znuA* gene, which showed potential positive selection with a posterior probability of 0.944 and a Bayes factor of 19.7. Site-specific analysis with SLAC did not detect any significant pervasive positive or negative selection, and no homoplasy event was identified in five of the seven genes (*greA* and *tolR* could not be evaluated due to the low number of informative characters).

Moreover, we compared the seven-loci MLST genes with the 2,289 resting cgMLST scheme genes across multiple analyses. MLST genes were significantly shorter than the cgMLST ones, a feature needed for Sanger sequencing, and showed significantly lower variability in terms of segregation sites, nucleotide diversity, and haplotype number (*p* = 0.044, *p* = 0.048, and *p* = 0.019, respectively). Simpson’s index analysis confirmed the robustness of the seven-loci MLST scheme compared to the cgMLST scheme, highlighting how the resolution level of the seven-loci MLST scheme is higher than the resolution level of single-linkage hierarchical clustering at 250 mismatches but lower when compared to the one of single-linkage hierarchical clustering at 100 mismatches (Supplementary Dataset 2). Tajima’s D test was carried out for all cgMLST genes, and the results indicated no strong selective pressure on the seven MLST genes, apart from *yciA,* which had a Tajima’s D value of −1.36, suggesting some degree of purifying selection (Supplementary Dataset 1).

### *In silico* assessment and *in vitro* evaluation of the *Providencia stuartii* MLST

To validate the discriminative power of our newly devised MLST scheme, we blind-tested it on an additional dataset composed of 24 *P. stuartii* genomes, downloaded between 1 June 2022 and 23 June 2023, which were not used to develop the scheme (marked by a * in Supplementary Table S2).

First, we performed a BLASTn analysis of the 7 designated genes to assign the respective alleles, which identified 9 different profiles in the 25 genomes from the additional dataset. While 7 of these profiles coincided with previously defined STs, 2 were unmatched and were associated with novel STs (named ST23 and ST24).

**Figure 2:**
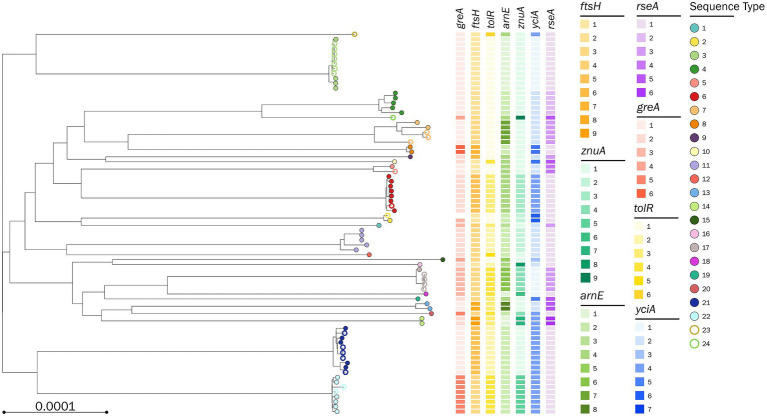
Phylogenetic tree based on a core-genome alignment of 71 *Providencia stuartii* genomes. The tips are color-coded according to the assigned sequence type (ST); full circles represent the genomes used to define the 7-loci multilocus sequence type (MLST); empty circles represent the genomes used to validate the 7-loci MLST; the allelic variants defining the MLST combination are color-coded according to the legend.

A second *P. stuartii* SNP-based phylogenetic tree was built starting from the 76 genomes (51 from the first dataset and 25 from the validation dataset), and the 7-loci MLST correctly predicted the core-genome distribution of the isolates assigned to each ST. Compared to the 51-genome tree (Supplementary Figure S1), the updated 76-genome tree displayed two novel branches, which corresponded to the genomes typed as ST23 and ST24 (Figure 2; Supplementary Table S2).

**Figure 3:**
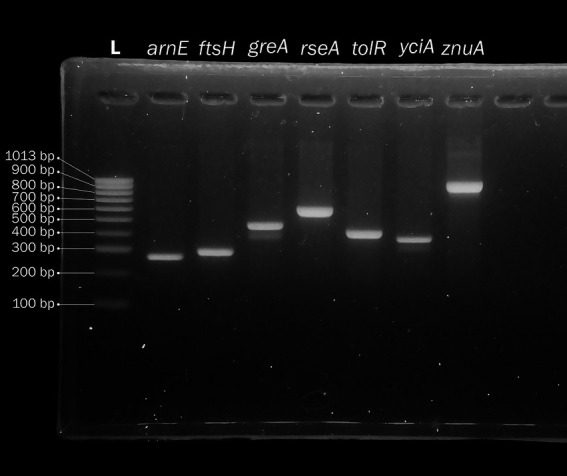
Agarose gel electrophoresis of a DNA ladder (lane 1) and the PCR amplification product of the seven genes used to define the *Providencia stuartii* multilocus sequence type (MLST, lanes 2–8).

To make the *P. stuartii* MLST independent from WGS, we also devised a set of PCR primers to amplify and sequence the seven housekeeping genes (Supplementary Table S1) and tested both amplification and Sanger sequencing procedures using strain 41 as a template (Figure 3).

## Conclusion

The current taxonomic classification of the *Providencia* genus is marked by substantial disarray. While a subset of our findings corroborates previous reports of incorrect reference assignments, within this manuscript, we did not attempt to resolve these issues. The focus of our study, instead, was to offer a robust framework for the identification and typing of the emerging species *P. stuartii*. Future research should aim to address and rectify the broader taxonomic ambiguities within the *Providencia* genus to enhance our understanding and management of these pathogens.

This study underscores the importance of *P. stuartii* as an emerging pathogen and provides a method for its accurate identification and typing through the development of MLST and cgMLST schemes.

Of note, to develop the 7-gene MLST scheme, we made use of a reverse approach: We first performed a core-genome-based phylogenetic analysis of *P. stuartii* genomes, which served as a cornerstone to define the STs, and then, we found the genes whose alleles fitted our *a priori* assignment. This approach could be applied to swiftly develop 7-gene MLST schemes for emerging species that cannot be typed with portable and scalable methods.

## Data Availability

A BioProject has been released at DDBJ/ENA/GenBank, No: PRJNA948429. Circular complete genome and plasmid of isolate 41 have been released under Acc. Nos. CP142095–CP142096. Reference *Providencia stuartii* NCBI RefSeq assembly no. GCF_029075745.1, isolate ATCC 29914. Single-nucleotide polymorphism (SNP) core-genome distance matrix of the 51 genomes used for the construction of the *Providencia stuartii* multilocus sequence typing scheme is supplied as Supplementary Dataset 1.

## Footnotes

^1^https://www.ncbi.nlm.nih.gov/data-hub/taxonomy/586/

^2^https://github.com/kblin/ncbi-genome-download

^3^https://github.com/donovan-h-parks/CompareM

^4^https://github.com/tseemann/snp-dists

## Supplementary material

SUPPLEMENTARY FIGURE S1 Phylogenetic tree based on a core-genome alignment of the 51 Providencia stuartii genomes used to define the first 22 sequence types (STs).

SUPPLEMENTARY FIGURE S2 Bandage (Wick et al., 2015) representation of the closed genome of the Providencia stuartii isolate 41 (Capitani et al., 2023). The position of the 7 genes chosen for the MultiLocus Sequence Type (MLST) definition is represented by colored, thick rectangles; the position of the 8 genes discarded is represented by grey, thin rectangles.

SUPPLEMENTARY DATASET Single Nucleotide Polymorphisms (SNPs) core-genome distance matrix of the 51 Providencia stuartii genomes used to define the relative MultiLocus Sequence Type (MLST) scheme. Genomes displaying less than 1,100 SNPs on the core genome are defined as belonging to the same Sequence Type (ST) and are highlighted in green.
