# Supplementary figures and images for: Development of a Providencia stuartii multilocus sequence typing scheme
Source: Front Microbiol. 2024 Oct 31;15:1493621. doi: 10.3389/fmicb.2024.1493621 (PMC11560872; doi:10.3389/fmicb.2024.1493621)

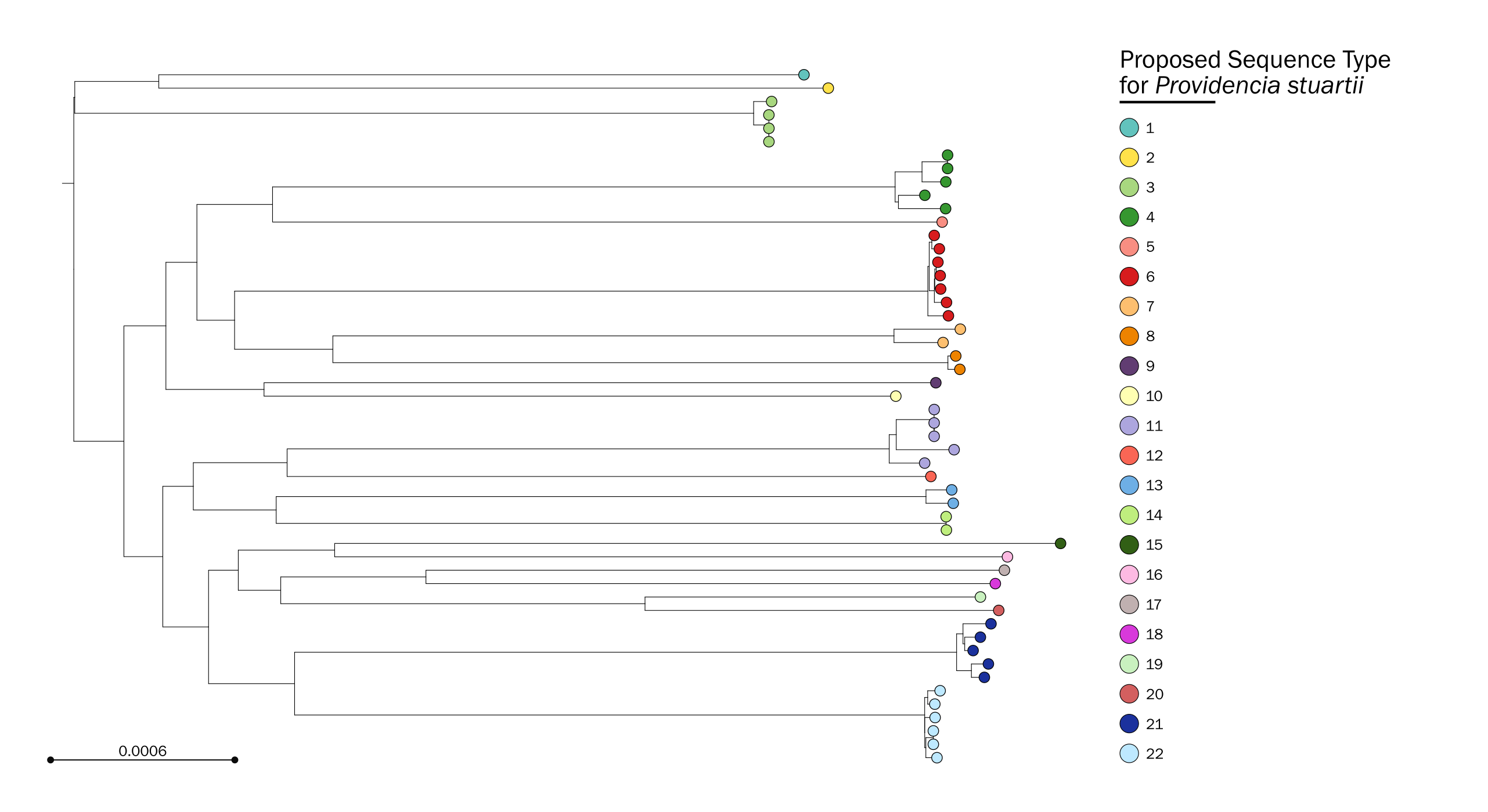

Supplement: Supplementary file 1 [file Image_1.png]

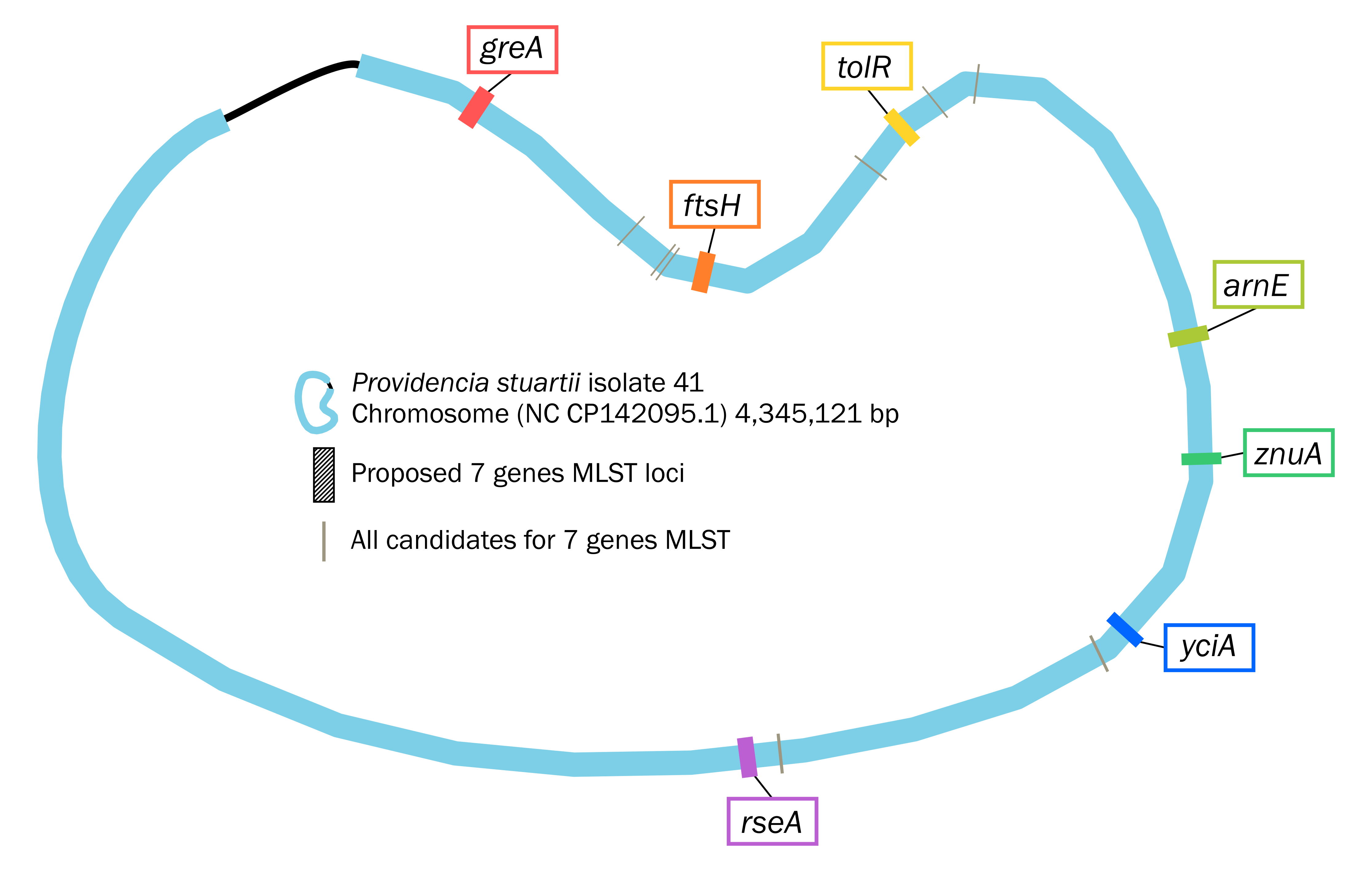

Supplement: Supplementary file 2 [file Image_2.png]
