# Supplementary material for: Development of a Providencia stuartii multilocus sequence typing scheme
Source: Front Microbiol. 2024 Oct 31;15:1493621. doi: 10.3389/fmicb.2024.1493621 (PMC11560872; doi:10.3389/fmicb.2024.1493621)
Supplement: Supplementary file 4 [file Table_2.docx]

**Supplementary Table 1:** Amplified genes, reaction conditions, primer pairs and Sanger sequencing allele templates used for the PCR amplification of the *Providencia stuartii* MLST genes

**Amplified genes**

**GreA** (transcription elongation factor)

**FtsH** (protease involved in the cell division process)

**TolR** (stator protein of the Tol-Pal a system)

**ArnE** (4-amino-4-deoxy-L-arabinose transporter)

**ZnuA** (Zn^++^ transporter)

**YciA** (acyl-CoA thioesterase)

**RseA,** (anti-sigma-E factor)

**Reaction conditions**

**DNA polymerase activation**

95 C 2 minutes

**29 cycles**

- **Denaturation:** 94 C 25 seconds
- **Annealing:** 55 C 25 seconds
- **Extension:** 72 C 1 minute and 15 seconds

**Amplification primer pairs**

**arnE_fw** GTA TGC TGG CAG GGT GAT AA 57,3 °C

**arnE_rv** TCA TCC GCT TAT ACT CAT CAA RAA 56,7 °C

**ftsH_fw** ATG AAT AAA AGC AAG CGC CTA TTA T 56,4 °C

**ftsH_rv** TTA TTT TTT ACC CGT ATT AAC AAC GC 56,9 °C

**greA_fw** ATG AAA CAG ATT CCA ATG ACG GT 57,1 °C

**greA_rv** TCA GAT ATA CTC AAC TTT AAG AAT TTC G 57,8 °C

**rseA_fw** AGA AAC TTT CCG CAA TGA TGG A 56,5 °C

**rseA_rv** TTA GCG CGC TTT AGC TCC A 56,7°C

**tolR_fw** ATG GCG CGC AAT AGT CGC 58,2 °C

**tolR_rv** TTA CAT GGG TTG AGT CAT TAA ACC 57,6 °C

**yciA_fw** TTC TAC GTA CTT TAG CTA TGC CT 57,1 °C

**yciA_rv** TAA CTG AAA ATG CTG TTT CTC TTT TG 56,9 °C

**znuA_fw** ATG ATA CAT AAA TCT CAA TTC ART GC 56,1 ° C

**znuA_rv** TTA ATT CAG GCA GCT CGC GTA 57,9 ° C

**Sequencing primer pairs**

**arnE_fw** GTA TGC TGG CAG GGT GAT AA 57,3 °C

**arnE_rv** TCA TCC GCT TAT ACT CAT CAA RAA 56,7 °C

**ftsH_fw** ATG AAT AAA AGC AAG CGC CTA TTA T 56,4 °C

**ftsH_rv** TTA TTT TTT ACC CGT ATT AAC AAC GC 56,9 °C

**greA_fw** ATG AAA CAG ATT CCA ATG ACG GT 57,1 °C

**greA_rv** TCA GAT ATA CTC AAC TTT AAG AAT TTC G 57,8 °C

**rseA_fw** AGA AAC TTT CCG CAA TGA TGG A 56,5 °C

**rseA_rv** TTA GCG CGC TTT AGC TCC A 56,7°C

**tolR_fw** ATG GCG CGC AAT AGT CGC 58,2 °C

**tolR_rv** TTA CAT GGG TTG AGT CAT TAA ACC 57,6 °C

**yciA_fw** TTC TAC GTA CTT TAG CTA TGC CT 57,1 °C

**znuA_fw** ATG ATA CAT AAA TCT CAA TTC ART GC 56,1 ° C

**znuA_rv** TTA ATT CAG GCA GCT CGC GTA 57,9 ° C

**znuA_seq_1** CGC ATA ATC ATG GGG TGA TG (additional sequencing primer)

**znuA_seq_2** GGC AGA ACA AGC AAA TAT TAT TCC (additional sequencing primer)

**Sanger sequencing**

Use the PCR primers also for sequencing.

For *znuA* two additional sequencing primers (znuA_seq_1 and znuA_seq_2), nested inside the PCR amplicons, are used together with the forward and reverse PCR primers.

**Allele templates**

**arnE** (**348 bp**)

ATGATTGCTCAATTATTATTACTGCTTTTAGTTAGTGTACTCACTTGCCTTGGGCAAATAGCGCAAAAGCAAGCGGTGGTATGCTGGCAGGGTGATAAACATAATAAAACGGCATCAGCCATTCGTTGGCTGGTGCTGGCATTGCTAATGCTTGGGTTAGGCATGCTGTTTTGGCTTAAATTGCTCGAAAGCATGCCGCTTAGCATTGCGTATCCAATGCTTAGTATTAATTTTGTGTTGGTTACTTTGATTGGTCAGTTTGTCTACCATGAGCAGACAGGGCTTAATCATTGGCTAGGTGTGGCGGCAATTATGTTTGGTATTTTCTTGATGAGTATAAGCGGATGA

**ftsH** (**300 bp**)

ATGAATAAAAGCAAGCGCCTATTATTGGTTAAAAAGAAACAGCGTTTAATCAAACAAATTGAGCAACAGCGTTCTGAACTCTCTGCGGCTTCCAAAGATTGGTTACAGATGACAGAGCCTTACGATCGCTCATGGCAAATCTTTGTCACCTTCCGGCCTATTTTTGTTGCCGCTGCCGGTCTTATTTCTCTTTATACCATTAAACGGCCTCAGCGTATTTTTTCCCTAGGCAAAAAAGCGATTGCCGCTTGGAGTTTAGTGCGAACCCTCCAAGGCGTTGTTAATACGGGTAAAAAATAA

**greA** (**477 bp**)

ATGAAACAGATTCCAATGACGGTATTGGGTGCAGATAAGTTACGAGAAGAGCTCGATTATCTTAAATCTGTCCGCCGCCCGGAAATTATTGCAGCGATTGCGGAAGCCCGCGAACACGGTGACTTGAAAGAGAACGCAGAATATCATGCCGCTCGCGAACAACAGGGNTTTTGTGAAGGCCGAATTCAAGAAATTGAATCCAAGCTTTCCCATGCTCAGGTTATTGACGTCACGAAAATGACAAACAATGGTCGTGTGATTTTTGGTGCGACAGTTACTGTGTTGAATGTTGATACGGATGAAGAGCTGACTTATCGCATTGTGGGTGATGATGAAGCAGATATTAAAATCAATCTGATTTCCGTTAACTCACCGATTGCACGCGGCTTGATCGGTAAAGAACAGGATGATGTTGTCACTATCAAAACGCCCGGTGGCGACGTTGAGTTCGAAATTCTTAAAGTTGAGTATATCTGA

**rseA** (**627 bp**)

ATGCAAAGAGAGAAACTTTCCGCAATGATGGATGGCGAAGTTCTAGATGTAGAACTGTTAAATGCCATTTCATGTGATTCAACGTTACAAAAACGTTGGGAGAGCTATCATCTTATTCGTGATACTCTGCGTAATGACACACCTGATGTCATCAATTTTGACATAGCGGGTAAAGTCGCCGCAGCATTAGAGAATGAAGTTGTGCGTATTAATCCTCAGGTAGTTGTTGAGTCACAGCCTGAACCTGCAACATGGGGCGCGATGCCGTTCTGGCAAAAAATTCGTCCATGGGCAAGTCAAATCACCCAAATTGGTGTTGCAGCATGTGTATCCCTTGCTGTTATCGTTGGGGTGCAGCAGTATAATCAAAGTAATTCAACGGAATCGGTGATTGATGAACCTGTGTTTAACACCGTTCCCGTTGGAGCAGGCGCACCTGTGAGTTTAAATTTCTCTGATGGTCAACTTTTTGGTAACGAGCAACAAATGCAACAAGTTGAGCAGCAAAATCAGCGTGTAAATGCAATGCTACAGCAATATGAAATTGAAAGACGTTCAATGCTTAATCAGCAATATCATGATAATGATGATATTACTACAGCACCCGCTGGAGCTAAAGCGCGCTAA

**tolR** (**423 bp**)

ATGGCGCGCAATAGTCGCAGACGTGAGCTAAAATCCGAAATCAACATTGTTCCATTGCTGGATGTTTTGTTGGTACTGTTGCTAATTTTTATGGCGACAGCACCGATTATTTCACAAAGCGTGGAGGTTGATCTGCCGGATGCCGCTGAAAGCCAGACCGTATCATCAAGCGATAATCCACCGATTATTTTAGAGGTTGCGGGAGTTGGTCAATACAATATGTTAATTGATGGTGAGCGCTTAGAGCTACTACCACCAGAACAGATTGCGGCTGAAGCGAAAGCTCAGCTAGATAAAAATCCAAAGGCGATTTTCCTTATCGGTGGTGCAAAAGAAGTGCCCTATGATGAAGTGATTAAGGCATTGAATATTTTGCATCAAGCTGGGATCAAATCGGTCGGTTTAATGACTCAACCCATGTAA

**yciA** (**417 bp**, use only yciA_fw for sequencing)

ATGCAATTACCAAACGGGGAATTAGTTCTACGTACTTTAGCTATGCCTGCCGATACAAATGCAAATGGGGATATTTTTGGCGGCTGGCTAATGTCTCAAATGGATATAGGTGGCGCGATATTAGCCAAAGAGATCGCGCTGGGTCGTGTGGTTACTGTGGCGGTGAATGGTATTAAATTTCAAAAGCCCGTCGCCGTAGGTGATGTGGTATGTTGTTACGCTCGTTGCTTAAAAACGGGCAAAAGCTCCATTACCATTAATATTGAAGTATGGGTTAAAAAAGTGGCAACGGAGCCTGTAGGCCATCGTTATCGTGCAACCGATGCCGTTTTTACCTATGTTGCCGTTAATGATGATAATACACCTCGTGAACTGCCAAAAGAGAAACAGCATTTTCAGTTAGAAAGCACTGAATAG

**znuA** (**960 bp**)

ATGATACATAAATCTCAATTCAATGCCCGACAATTTATTTTAGGCNCCGTTGCGGCATCTGTCCTTAGCACAACGTTGGTCACTGCGGCAAAGGCGGATGTTGTGACCTCTATTCGCCCACTTGCCTTTATTGCCGCGGGTATCGCTGATGGTGTGACTGATACTCAGGTCTTGCTGCCAGATGGTGCATCACCCCATGATTATGCGCTTAAACCTTCTGATTTGAGGAAAATTAAACAGGCAGATCTTTTTGTTTGGGTAGGCCCCGATATGGAGATGTTTTTAGATAAGCCACTCAAGACGTTACCACAGGATAAACGTCTGGCGCTGGCAGAACAAGCAAATATTATTCCCTTGCTGATGAAAGGGGATGAGGAAGAGGCGCATGACCATGAACAGGGTGATGAAGCTAATCATGATGAGGATCACGAACATCACCACCACGGTGAGTATAATATGCATATTTGGCTGTCGCCCGAGATTGCAAAACAGGCTGCGTTAGACATCCACGATCGTTTAGTAGTGCTTTATCCTGACCAAAAAGATAAGCTGGACGTAAACCTTCGTAAATTCAATGAAAAAATGACGCAAAACGATAAGAAAATTGTTAATATTTTAGAACCAGCCAAAAACAAGGGCTATTTTGTTTTTCACGATGCCTACGGCTACTTTGAAAAACACTATCGGTTGGCTCCTTTGGGGCATTTTACGATAAACCCGGAAATTCAGCCTGGTGCACAGAAATTACATAAAATACGAACTCAATTGGTTGAGCATAAAGCACAATGTGTTTTTGCTGAACCCCAATTCAGGCCAGCCGTCATAGAGAGCGTAGCAAAAAATACGGGAGTCAAAATGGGAACTCTCGACCCGCTAGGTAGCGGGCTGGAAATAGGACCAGATAGCTATATGCAGTTTTTAACGCAACTGTCTAAGCAATACGCGAGCTGCCTGAATTAA
