# Supplementary material for: Development of a Providencia stuartii multilocus sequence typing scheme
Source: Front Microbiol. 2024 Oct 31;15:1493621. doi: 10.3389/fmicb.2024.1493621 (PMC11560872; doi:10.3389/fmicb.2024.1493621)
Supplement: Supplementary file 5 [file Table_3.docx]

**Supplementary Table 2**: Allelic profiles and genomes of the 7 loci used to define the 24 different *Providencia stuartii* MultiLocus Sequence Type (MLST) groups.

* Genomes used for the *P. stuartii* MLST validation

| **Sequence Type** | ***greA, ftsH, tolR, arnE, znuA, yciA, rseA*** | **Genome name** |
| --- | --- | --- |
| 1 | 3, 4, 3, 3, 1, 2, 3 | GCF_001267785 |
| 2 | 3, 1, 1, 2, 2, 7, 1 | GCF_017152395 |
|  |  | GCF_000783455* |
| 3 | 1, 1, 1, 1, 1, 1, 1 | 41 |
|  |  | 65 |
|  |  | 883 |
|  |  | GCF_000259175 |
|  |  | GCF_028477805* |
|  |  | GCF_028478105* |
|  |  | GCF_029075645* |
|  |  | GCF_029075685* |
|  |  | GCF_029075745* |
|  |  | GCF_029075755* |
|  |  | GCF_029075765* |
| 4 | 1, 2, 1, 2, 1, 2, 2 | GCF_000154865 |
|  |  | GCF_009706595 |
|  |  | GCF_009706655 |
|  |  | GCF_018413475 |
|  |  | GCF_902373775 |
| 5 | 1, 1, 1, 4, 1, 2, 4 | GCF_001463005 |
| 6 | 2, 5, 3, 4, 3, 4, 1 | GCF_001888205 |
|  |  | GCF_002983665 |
|  |  | GCF_015739445 |
|  |  | GCF_015739495 |
|  |  | GCF_015739565 |
|  |  | GCF_015739585 |
|  |  | GCF_900455285 |
|  |  | GCF_028482435* |
| 7 | 1, 3, 1, 7, 1, 2, 3 | GCF_003688015 |
|  |  | GCF_009706265 |
|  |  | GCF_028482385* |
|  |  | GCF_028482365* |
| 8 | 6, 7, 1, 2, 1, 6, 1 | GCF_008693805 |
|  |  | GCF_010597545 |
| 9 | 2, 6, 1, 4, 1, 2, 3 | GCF_001968995 |
| 10 | 2, 1, 4, 4, 1, 6, 5 | GCF_023520575 |
| 11 | 2, 3, 2, 2, 2, 2, 1 | GCF_000754345 |
|  |  | GCF_009706605 |
|  |  | GCF_015739395 |
|  |  | GCF_016128115 |
|  |  | GCF_016623625 |
| 12 | 2, 3, 5, 2, 1, 2, 1 | GCF_009706235 |
| 13 | 1, 8, 3, 8, 1, 2, 5 | GCF_009706215 |
|  |  | GCF_014652175 |
| 14 | 2, 9, 3, 1, 7, 4, 6 | GCF_013166725 |
|  |  | GCF_013166755 |
| 15 | 4, 3, 1, 5, 1, 3, 1 | GCF_001558855 |
| 16 | 2, 1, 2, 4, 8, 2, 1 | GCF_015739525 |
| 17 | 3, 3, 4, 6, 4, 1, 3 | GCF_002206175 |
|  |  | GCF_029075585* |
|  |  | GCF_029075605* |
|  |  | GCF_029075615* |
|  |  | GCF_029075875* |
| 18 | 3, 3, 4, 1, 6, 1, 1 | GCF_010589975 |
| 19 | 2, 3, 3, 4, 1, 5, 5 | GCF_002886955 |
| 20 | 5, 3, 4, 2, 4, 4, 1 | GCF_010588105 |
| 21 | 1, 5, 2, 2, 1, 4, 1 | GCF_002947315 |
|  |  | GCF_010669105 |
|  |  | GCF_012956045 |
|  |  | GCF_015832305 |
|  |  | GCF_017153315 |
|  |  | GCF_028477385* |
|  |  | GCF_028477885* |
|  |  | GCF_028477905* |
|  |  | GCF_028477925* |
|  |  | GCF_028482395* |
| 22 | 5, 3, 4, 2, 5, 4, 1 | GCF_006517525 |
|  |  | GCF_010320365 |
|  |  | GCF_017152355 |
|  |  | GCF_017152365 |
|  |  | GCF_017152445 |
|  |  | GCF_023572545 |
|  |  | GCF_030179155* |
|  |  | GCF_029350705* |
| 23 | 1, 1, 6, 4, 1, 4, 1 | GCF_028480065* |
| 24 | 4, 3, 1, 4, 9, 2, 5 | GCF_028477825* |
